# Case Report: Can preoperative implantation of veno-arterial extracorporeal membrane oxygenation lead to embolic events in infective endocarditis?

**DOI:** 10.3389/fcvm.2024.1334457

**Published:** 2024-03-28

**Authors:** Tianlong Li, Xiaoxiao Wu, Tingrui Chen, Chun Pan, Ruiming Yue, Chunlin Xiang, Tao Yu, Zhenjie Jiang, Xiaobo Huang, Xuemei Tang, Yiping Wang

**Affiliations:** ^1^ Department of ICU, Sichuan Provincial People’s Hospital, University of Electronic Science and Technology of China, Chengdu, China; ^2^ Medical School, University of Electronic Science and Technology of China, Chengdu, China; ^3^ Department of Cardiac Surgery, Sichuan Provincial People’s Hospital, University of Electronic Science and Technology of China, Chengdu, China

**Keywords:** infective endocarditis, extracorporeal membrane oxygenation, cardiogenic shock, embolism, multidisciplinary team (MDT)

## Abstract

Early-stage infective endocarditis (IE) can lead to severe complications, including infarctions and metastatic infections caused by inflammatory embolus shedding. Common embolism sites include the brain, spleen, kidneys, lungs, and intestines. Additionally, acute heart failure (AHF) can occur in up to 40% of cases, and its presence can impact the clinical outcomes of patients with IE. Cardiogenic shock (CGS) is often more likely to occur after AHF has taken place. If bacteria invade the blood, infectious shock can occur. Patients with IE can experience simple CGS, septic shock, or a combination of the two. Extracorporeal membrane oxygenation (ECMO) typically serves as a Bridge for Heart failure and Cardiogenic shock. Previous research indicates that there are limited reports of ECMO support for patients with IE after CGS has occurred. Because CGS may occur at any time during IE treatment, it is important to understand the timing of ECMO auxiliary support and how to carry out comprehensive treatment after support. Timely treatment can help to reduce or avoid the occurrence of serious complications and improve the prognosis of patients with IE. Our work combines a case study to review the ECMO support of IE patients after CGS through a literature review. Overall, we suggest that when patients with IE have large bacterial thrombosis and a greater risk of shedding, it is recommended to carefully evaluate the indications and contraindications for ECMO after discussion by a multidisciplinary team (MDT). Still, active surgical treatment at an early stage is recommended.

## Introduction

Infective endocarditis (IE), also known as bacterial endocarditis, is an infection caused by bacteria that enter the bloodstream and settle in the heart lining, a heart valve, or a blood vessel. This infection has a high incidence and mortality rate (1). Complications are often caused by infarcts and metastatic infections due to inflammatory embolus shedding. Common embolic sites include the brain, spleen, kidneys, lungs, and intestines. Among all sites, neurological complications are the most common and serious extracardiac complications (2). Acute heart failure (AHF) occurs in 19%–44% of cases (3, 4), and it can seriously affect the clinical outcomes of patients with IE. Although previous research has been conducted on IE and extracorporeal membrane oxygen (ECMO) for mechanical circulatory support, information is limited on the diagnosis and treatment of patients with IE after cardiogenic shock (CGS) has occurred.

This report describes the first case of a patient who showed both AHF and CGS at the time of consultation and received veno-arterial extracorporeal membrane oxygenation (VA-ECMO) auxiliary support while in emergency care. However, there was eventually necrosis of the nasal alar and both lower limbs, along with multiple intracranial and spleen infarctions. Our work, combined with a comprehensive literature review, reveals how ECMO techniques can reduce or avoid strategies for multiple embolism and even death when applied to similar patients.

## Case report

**Figure 1:**
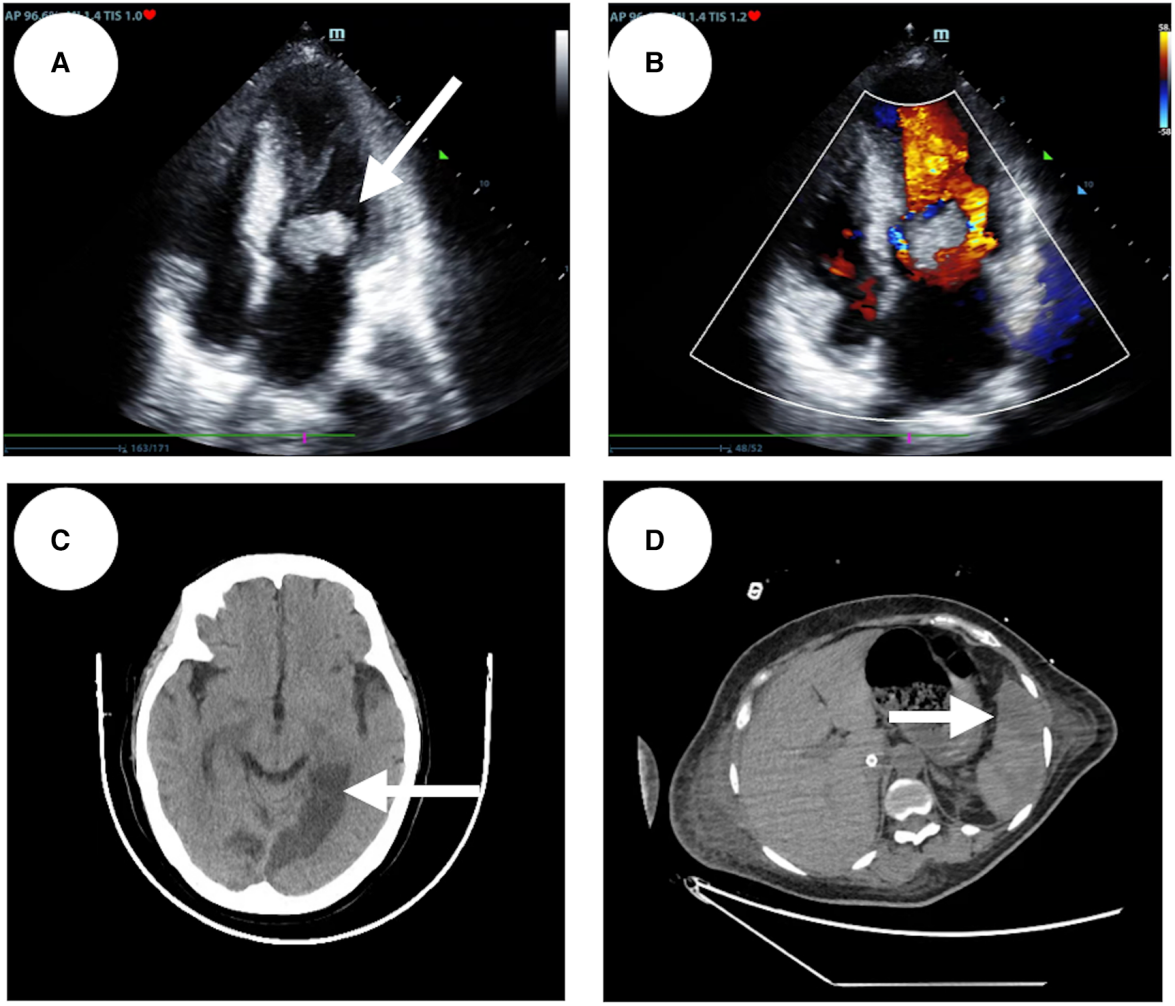
Patient's emergency echocardiography, abnormal CT scans of the patient's head and abdomen. (**A**) The patient's apical four-chamber view of echocardiography. (**B**) An irregular isoechoic mass of approximately 45 × 27 mm in size was attached to the anterior mitral valve (indicated by the white arrow). (**C**) On the left side of the anterior horn of the lateral ventricle, the left thalamus, the left parietal-occipital lobe, and the left side of the splenium of the corpus callosum, a patchy low density shadow with clear boundaries is visible (indicated by the white arrows). (**D**) Patchy low-density shadow (indicated by the white arrows) on the spleen.

**Figure 2:**
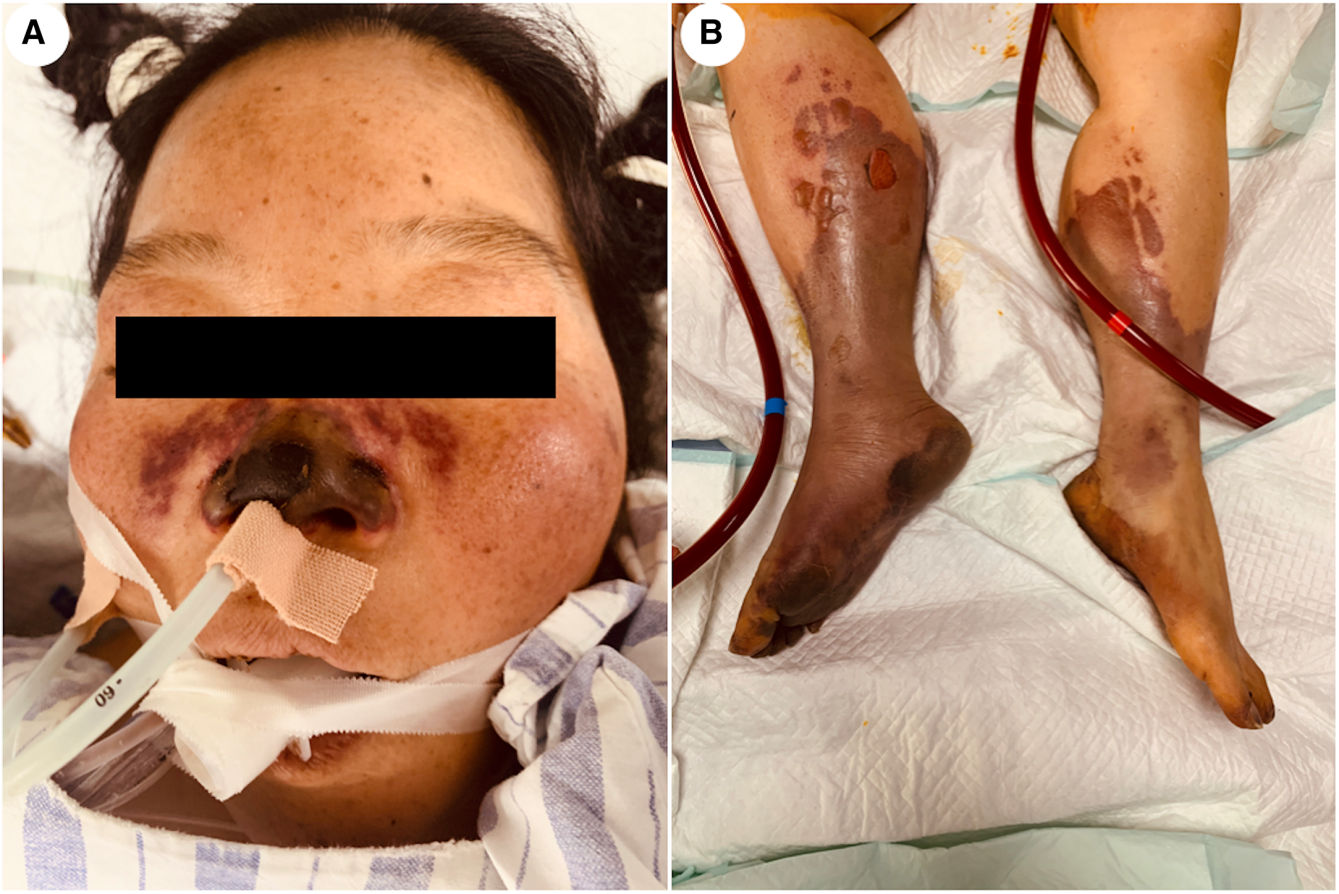
Patient's facial and lower limb skin. (**A**) The tip of the patient's nose and both sides of the nose are partially dark. (**B**) The skin on both lower limbs is swollen with a bluish color.

A 48-year-old woman was admitted to the hospital with the chief complaint of general weakness and cardiac tiredness for over two months. The patient was also aggravated with somnolence for 8 days. Previously, the patient was in good health and had no special medical history, family history, or social psychological history. The color Doppler ultrasound examination at the local hospital showed normal cardiac structure and function. However, her condition worsened over time. She appeared to be unconscious, with cold limbs and skin mottling on her thighs. The doctor established an artificial airway for ventilator treatment and quickly treated the patient with 2,000 ml of crystalloid solution; her arterial blood pressure was only 78/39 mmHg (norepinephrine 1.2 ug/kg min^−1^), and pink foam sputum could be aspirated from the airway. She was eventually transferred to the Municipal People's Hospital. An emergency echocardiogram showed irregular and isoechoic mass attachment in the anterior leaflet of mitral valve, with a size of about 45 × 27 mm (Figure 1) and EF of 30%. The patients’ blood pressure was still progressively decreasing, and the arterial blood gas results showed a pH of 7.10 and lactate of 15 mmol/L. She was given peripheral VA-ECMO, fluid infusion, vasoactive agents, and antibiotics. Subsequently, her piebald lower limb condition improved. Due to the lack of extracorporeal circulation surgery facilities at the local hospital, she was transferred to our hospital the next afternoon for further treatment. Upon admission, the patient's VA-ECMO blood pressure was maintained at 79/61 mmHg (ECMO flow 3.4 L/min, norepinephrine 0.3 ug/kg min^−1^). Additionally, she was unconscious and the tip and both sides of the nose were partially dark (Figure 2). Cardiac auscultation showed a systolic rumbling murmur in the auscultation area of the mitral valve, as well as coolness, swelling, and cyanosis of both lower limbs (Figure 2). The right leg circumference was larger than that of the left and the pulse of the bilateral dorsalis pedis arteries was not palpable. The echocardiogram showed that there was a neoplasm in the front valve of the mitral valve with a size of about 15 × 12 mm. This measurement was smaller than the local hospital's result. A venous ultrasound of both lower limbs also showed venous thrombosis in the right lower limb. CT examination showed multiple cerebral infarction and splenic infarction (Figure 1). A full blood cell analysis revealed a white blood count of (WBC) 19.710 × 10^9 ^/L, neutrophil rate 95.0%, HGB 87 g/L, Plt 14 × 10^9 ^/L; and BNP 1,552.0 pg/ml; as well as MYO 9,528.7 ng/ml, hypersensitivity troponin I 154,579.2 ng/L; DIC: PT 16.6 s, APTT 74.3 s, and D-dimer > 64.00 mg/L FEU. The final diagnoses were: (1) IE, (2) acute heart failure, (3) cardiogenic shock, (4) splenic infarct and cerebral infarction, and (5) necrosis of both lower limbs, nasal necrosis, and terminal finger necrosis of both hands. On the same day, the multidisciplinary team (MDT) considered that the patient had an emergency surgery indication, but CT indicated that multiple infarctions were likely caused by thrombus shedding. Therefore, the patient's long-term prognosis was poor, and the risks of surgery was extremely high. Her family decided to forgo treatment and the patient died on the same day after she was discharged. The following day, blood NGS results showed the presence of Streptococcus mitis (Table 1). and the blood culture result was negative.

**Table 1:** The patient's blood NGS results indicated the presence of *Streptococcus mitis.*

Types	Species			Category	
	Latin name	Sequence number	Relative abundance	Latin name	Sequence number
G^+^	*Streptococcus*	711	88.65%	*Streptococcus mitis*	469
				*Streptococcus pneumoniae*	99
G^−^	*Enterococcus*	7	0.87%	*Enterococcus faecium*	7

## Discussion

AHF is the most common and serious complication of active IE, causing an increased risk of death (5, 6). Extracorporeal membrane oxygenation (ECMO) is an artificial *in vitro* support system (7). By circulating the patients’ blood through an artificial “lung” or “membrane, the system delivers oxygen and removes carbon dioxide to meet the patient’s metabolic needs. ECMO can be applied to patients with cardiac arrest, acute severe heart failure, acute severe respiratory failure, or other serious threats to respiratory and circulatory functions. However, IE is not a common contraindication of ECMO. Our literature review of IE and ECMO for mechanical circulatory support resulted in a total of 10 articles. Nine of them were case reports and one was a retrospective case study describing patients requiring ECMO auxiliary support during the treatment of IE (Table 2). Among the patients studied, a majority were supported by ECMO following surgery and the prognosis was generally positive (9–11, 13). In the case reports in which patients received ECMO support before surgery, individual cases were also successful (12, 14, 16, 17). The only multicenter retrospective case study was published by Handa K et al. (8), in which two patients needed ECMO support before surgery due to cardiogenic shock and 10 patients needed ECMO support due to postoperative cardiogenic shock. The final prognosis of these patients was not described in the article.

**Table 2:** Patients with IE requiring ECMO auxiliary support.

Literature	Type	Disease	Reason for ECMO	ECMO timing	Auxiliary time (day)	Timing of surgery	Operation	Complications	Prognosis
Handa et al. (8)	Retrospective multicenter study	IE	2 cases were preoperative cardiogenic shock and 10 cases were postoperative cardiogenic shock	2 cases before surgery, 10 cases after surgery	–	–	–	–	–
Edlin et al. (9)	Case	Austrian syndrome	Difficulty in separating from extracorporeal circulation	After CPB surgery	6	Emergency surgery	Aortic valve and mitral valve replacement	Watershed cerebral infarcts with hemorrhagic transformation, polycystic lung lesions, new mitral and aortic paraprosthetic leaks (PPLs)	Survived
Ibrahim et al. (10)	Case	Congenital heart disease, device-associated infective endocarditis	Cardiogenic shock	After CPB surgery	–	–	–	Multiple complications	Survived
Nagase et al. (11)	Case	Behçet's disease, mechanical aortic valve falling off	Difficulty in separating from extracorporeal circulation	After CPB surgery	5	Emergency surgery	Redo aortic valve replacement	Multiple intracranial infarction	Survived after three months of follow-up, unknown later
Noyes et al. (12)	Case	IE with aorto-atrial fistula	Rupture of the aortic valve, with severe aortic insufficiency	Before CBP surgery	1/3	Emergency surgery	Excision of valve, debridement of all vegetation	–	Survived
John et al. (13)	Case	IE	Case #1: refractory hypoxia. Case #2: biventricular failure. Case#3: refractory hypoxia	Case #1: unoperated. Case #2: after CPB surgery. Case #3: unoperated	Case #1:1 Case #2: not mentioned Case#3: 2	Emergency surgery	Case#1: unoperrated Case#2: aortic valve replacement Case#1: unoperrated	–	All died
Bainiwal et al. (14)	Case	Acute decompensated heart failure in the setting of severe bioprosthetic mitral valve stenosis	Pulseless elective activity (PEA) arrest	Before surgery	8	–	Trans-catheter mitral valve-in-valve (TMViV) replacement	–	Survived
Usui et al. (15)	Case	IE	Sudden cardiac arrest for ACS	Before CBP surgery	Not mentioned	Emergency surgery	Aortic and mitral valve replacement	Multiple organ failure	Died
Kunioka et al. (16)	Case	Cardiopulmonary arrest caused by embolic occlusion in the left main artery and infective endocarditis on the mitral valve	Pulseless electrical activity	Before CBP surgery	–	Emergency surgery	Percutaneous transluminal coronary angioplasty of the left main coronary artery and mitral valve replacement, extracorporeal left ventricular assist device	Coronary artery embolism	Survived
Chen et al. (17)	Case	Device infection, sepsis, bacterial pericarditis and heparin-induced thrombocytopenia	Non-ischemic dilated cardiomyopathy, acute lung injury.	Before CBP surgery	13	–	Orthotopic heart transplantation	–	Survived

When the patient we reported was transferred to the Municipal People's Hospital, high-dose vasoactive drugs were unable to maintain a normal blood pressure, and there was skin mottling on both thighs, as well as anuria. The arterial blood gas showed severe acidosis, with lactate of 15 mmol/L. Emergency echocardiography showed the formation of mitral valve vegetation, mitral valve insufficiency, and an EF of 30%. The patient was diagnosed with AHF and CGS, and they had a VA-ECMO surgical indication. After placement of the VA-ECMO system, the patient's condition improved for several hours, with norepinephrine reduced to 0.8 ug/kg min^−1^, arterial blood lactate reduced to 12.6 mmol/L, and bilateral thigh skin mottling disappearing. The patient showed low platelet levels and she did not use heparin anticoagulation. After admission to our hospital, the CT showed multiple brain and spleen infarctions, and the nasal tip displayed cyanochroia. There were no reports of nasal ischemia and necrosis due to severe shock and IE, but cases of cavernous sinus thrombosis caused by primary nasal infection have been previously reported (18). When observing the development of the patient's condition after receiving ECMO, the echocardiogram revealed that the size of the mitral valve Neoplasm was 15 × 12 mm, which was smaller than the 45 × 27 mm found in the local hospital. We speculated that the patient had experienced an embolic event and the possibility of bacterial embolus shedding was high. Unfortunately, there was no pathological result of nasal necrosis or pathogenic bacteria result to confirm our original assumption.

The risk of embolism events occurring within the first four weeks of IE treatment is very high, so it is necessary to treat with antibiotics early on. The patient did not use anticoagulants due to low platelet levels and eventually developed multiple embolisms throughout the body. Through our literature search, we found no related reports on whether this patient was suitable for ECMO and whether bacterial embolus shedding is related to it. However, according to previous reports (19), the probability of embolism events and death in patients with neoplasms larger than 10 mm increased compared to patients with IE who have neoplasms smaller than 10 mm in size. The patient's tumor was approximately 45 × 27 mm and was prone to detachment and vascular embolism.

Because general VA-ECMO blood flow in the aorta was reversed, there was an increased risk of mitral valve neoplasms falling out in patients with aortic insufficiency. This can increase the probability of embolic complications. Common ECMO complications in a patient's body include bleeding from surgical wounds and intubation sites, embolism, hemolysis, renal dysfunction, infection, and abnormal nervous system function (8, 20). Among them, the probable cause of embolism in ECMO patients is that ECMO flow is too high, leading to insufficient blood flow to the left ventricle. At the same time, the aortic valve also needs to overcome the pressure from ECMO reverse blood flow. Some patients may experience obstruction of the opening of the aortic valve and stagnation of blood flow in the left ventricle, which can form an adjoining wall thrombus. When the valve reopens, it may push the thrombus towards the distal end, leading to an embolic event. During this time, it is possible to reduce the probability of associated embolic events by adjusting the amount of anticoagulant, monitoring the left ventricular blood flow with echocardiography, and observing whether the aortic valve opens to guide the regulation of ECMO flow. In this case, due to abnormal coagulation function and low platelet counts, anticoagulants were not used. An ultrasound showed that the aortic valve was open, that there was no left ventricular blood stasis or thrombosis, and the possibility of thrombus detachment leading to infarction was extremely low. However, since the site of attachment of the neoplasm was on the mitral valve and there were larger valvular neoplasms with a high risk of shedding, we were not able to determine whether we promoted shedding of the neoplasm after applying ECMO.

In a review of the Osaka Cardiovascular Research (OSCAR) group database, Kazuma Handa et al. discovered that from 2009 to 2017, only two patients required VA-ECMO mechanical-assisted support (due to cardiogenic shock) before surgery, and 10 patients received ECMO support following surgery (8). The current guidelines recommend early surgical intervention for AHF patients, especially those with cardiogenic shock (21, 22). Further, most of the emboli of arterial embolisms caused by IE are bacterial thromboses; it is difficult to achieve blood vessel revascularization solely through thrombolytic and anti-coagulant medication for this condition. Typically, after the embolism event is diagnosed, the patient's overall condition should be immediately assessed. If the patient can withstand the operation, surgery should be initiated immediately.

## Conclusions

When patients are highly suspected of having experienced cardiogenic shock caused by IE and there is a risk of vegetative shedding, it is necessary to carefully evaluate the indications for ECMO. This is especially true for patients with IE who are at greater risk of neoplasm shedding. If there are patients with IE who have been placed with VA-ECMO, we need to dynamically observe the size and location of the neoplasm and perform emergency surgical treatment as soon as possible. If the treatment facility cannot support adequate conditions for surgery, the patient should be transferred to a facility that can perform surgery as soon as possible before serious complications arise. Naturally, this conclusion still requires further support from additional research data.

## Data Availability

The original contributions presented in the study are included in the article/Supplementary Materials, further inquiries can be directed to the corresponding authors.

## References

[1] Murdoch DR Corey GR Hoen B Miró JM Fowler VG Jr Bayer AS Clinical presentation, etiology, and outcome of infective endocarditis in the 21st century: the international collaboration on endocarditis-prospective cohort study. Arch Intern Med. (2009) 169(5):463–73. 10.1001/archinternmed.2008.603 19273776 PMC3625651

[2] García-Cabrera E Fernández-Hidalgo N Almirante B Ivanova-Georgieva R Noureddine M Plata A Neurological complications of infective endocarditis: risk factors, outcome, and impact of cardiac surgery: a multicenter observational study. Circulation. (2013) 127(23):2272–84. 10.1161/CIRCULATIONAHA.112.000813 23648777

[3] Kiefer T Park L Tribouilloy C Cortes C Casillo R Chu V Association between valvular surgery and mortality among patients with infective endocarditis complicated by heart failure. JAMA. (2011) 306(20):2239–47. 10.1001/jama.2011.1701 22110106 PMC5030065

[4] Nadji G Rusinaru D Rémadi JP Jeu A Sorel C Tribouilloy C. Heart failure in left-sided native valve infective endocarditis: characteristics, prognosis, and results of surgical treatment. Eur J Heart Fail. (2009) 11(7)668–75. 10.1093/eurjhf/hfp077 19553397

[5] Habib G Lancellotti P Antunes MJ Bongiorni MG Casalta JP Del Zotti F 2015 ESC guidelines for the management of infective endocarditis: the task force for the management of infective endocarditis of the European society of cardiology (ESC) Endorsed by: European association for cardio-thoracic surgery (EACTS), the European association of nuclear medicine (EANM). Eur Heart J. (2015) 36(44):3075–128. 10.1093/eurheartj/ehv319 26320109

[6] Bohbot Y Habib G Laroche C Stöhr E Chirouze C Hernandez-Meneses M Characteristics, management, and outcomes of patients with left-sided infective endocarditis complicated by heart failure: a substudy of the ESC-EORP EURO-ENDO (European infective endocarditis) registry. European J of Heart Fail. (2022) 24(7):1253–65. 10.1002/ejhf.2525 35508915 PMC9543970

[7] Hadaya J Benharash P. Extracorporeal membrane oxygenation. JAMA. (2020) 323(24):2536. 10.1001/jama.2020.9148 32463441

[8] Handa K Yoshioka D Toda K Yokoyama JY Samura T Suzuki K Surgical results for infective endocarditis complicated with cardiogenic shock. Circ J. (2020) 84(6):926–34. 10.1253/circj.CJ-19-0583 32295976

[9] Edlin JC Metwalli A Finney SJ Ambekar SG. Postcardiotomy extracorporeal membrane oxygenation in a patient with Austrian syndrome. BMJ Case Rep. (2020) 13(2):e233564. 10.1136/bcr-2019-233564 32114496 PMC7050310

[10] Ibrahim W Hoschtitzky A Thakuria L Li W Semple T Clague J Follow the lead: the challenges of cardiogenic shock in device-related infective endocarditis. JACC Case Rep. (2021) 3(9):1163–9. 10.1016/j.jaccas.2021.05.012 34401751 PMC8353571

[11] Nagase H Hoashi T Masuoka A Hotoda K Toda K Yoshitake A Completely detached mechanical aortic valve prosthesis stuck to the aortic arch in a patient with behçet’s disease. Surg Case Rep. (2022) 8(1):143. 10.1186/s40792-022-01506-6 35904703 PMC9338190

[12] Noyes AM Ramu B Parker MW Underhill D Gluck JA. Extracorporeal membrane oxygenation as a bridge to surgery for infective endocarditis complicated by aorto-atrial fistula and cardiopulmonary collapse. Tex Heart Inst J. (2015) 42(5):471–3. 10.14503/THIJ-14-4575 26504445 PMC4591891

[13] John SG William P Murugapandian S Thajudeen B. Outcome of patients with infective endocarditis who were treated with extracorporeal membrane oxygenation and continuous renal replacement therapy. Clin Pract. (2014) 4(3):670. 10.4081/cp.2014.670 25568769 PMC4274487

[14] Bainiwal JS Ham JA Aksoy O. Trans-catheter mitral valve-in-valve replacement in a patient on venoarterial extracorporeal membrane oxygenation: a case report. Eur Heart J Case Rep. (2023) 7(9):ytad427. 10.1093/ehjcr/ytad427 37719001 PMC10500414

[15] Usui R Mutsuga M Narita Y Tokuda Y Terazawa S Ito H Sudden circulatory collapse caused by mechanical obstruction of the left main coronary trunk with infective endocarditis vegetation: a case report. Surg Case Rep. (2021) 7(1):223. 10.1186/s40792-021-01296-3 34648077 PMC8517051

[16] Kunioka S Tadokoro N Fujita T Fukushima S. Successful exclusion of left main trunk coronary artery aneurysm and concomitant HeartMate 3 implantation in a patient with a history of infective endocarditis: a case report. Eur Heart J Case Rep. (2023) 7(3):ytad080. 10.1093/ehjcr/ytad080 36909834 PMC9994585

[17] Chen E Clarke N Huffman L Peltz M. Transplantation in a patient on extracorporeal membrane oxygenation with infective endocarditis, pericarditis and heparin-induced thrombocytopenia. Interact Cardiovasc Thorac Surg. (2017) 24(3):462–3. 10.1093/icvts/ivw359 28040771

[18] Nagarakanti S Bishburg E Brown M. Cavernous sinus thrombosis due to Streptococcus mitis and Staphylococcus lugdunensis. J Clin Diagn Res. (2016) 10(9):OD13–4. 10.7860/JCDR/2016/21521.8545 27790500 PMC5072000

[19] Mohananey D Mohadjer A Pettersson G Navia J Gordon S Shrestha N Association of vegetation size with embolic risk in patients with infective endocarditis: a systematic review and meta-analysis. JAMA Intern Med. (2018) 178(4):502–10. 10.1001/jamainternmed.2017.8653 29459947 PMC5876809

[20] Wrisinger WC Thompson SL. Basics of extracorporeal membrane oxygenation. Surg Clin North Am. (2022) 102(1):23–35. 10.1016/j.suc.2021.09.001 34800387 PMC8598290

[21] AATS Surgical Treatment of Infective Endocarditis Consensus Guidelines Writing Committee Chairs, Pettersson GB Coselli JS Committee W Pettersson GB Coselli JS 2016 the American association for thoracic surgery (AATS) consensus guidelines: surgical treatment of infective endocarditis: executive summary. J Thorac Cardiovasc Surg. (2017) 153(6):1241–1258.29. 10.1016/j.jtcvs.2016.09.093 28365016

[22] Baddour LM Wilson WR Bayer AS Fowler VG Jr Tleyjeh IM Rybak MJ Infective endocarditis in adults: diagnosis, antimicrobial therapy, and management of complications: a scientific statement for healthcare professionals from the American heart association. Circulation. (2015) 132(15):1435–86. 10.1161/CIR.0000000000000296 26373316

